# Diplopia as the herald of fulminant myocarditis in undifferentiated connective tissue disease: a case report

**DOI:** 10.3389/fcvm.2026.1848981

**Published:** 2026-06-04

**Authors:** Yongqi Chen, Yinping Li, Rui Zhang, Xiaokang Liu, Jingwen Zhang, Ning Zhai, Hanheng Zuo, Xueying Chen

**Affiliations:** 1 College of Clinical Medicine, Jining Medical University, Jining, China; 2 Department of Cardiology, Affiliated Hospital of Jining Medical University, Jining, China; 3 Department of Pathology, Affiliated Hospital of Jining Medical University, Jining, China; 4 Department of Medical Imaging, Affiliated Hospital of Jining Medical University, Jining, China

**Keywords:** undifferentiated connective tissue disease, fulminant myocarditis, diplopia, endomyocardial biopsy, case report

## Abstract

This manuscript describes a rare case of undifferentiated connective tissue disease (UCTD)-associated fulminant myocarditis presenting with isolated diplopia as the sole initial manifestation. A 66-year-old Chinese woman presented with a 17-day history of isolated diplopia. Initial evaluation revealed markedly elevated cardiac enzyme profile and electrocardiographic changes suggestive of acute myocardial infarction. However, coronary angiography revealed no coronary stenosis. Cardiac magnetic resonance imaging (MRI) was consistent with myocarditis. The initial diagnoses included Miller-Fisher syndrome and inflammatory cardiomyopathy. The patient underwent plasma exchange, intravenous methylprednisolone (20 mg daily), and intravenous immunoglobulin (IVIg). Her condition improved. After discharge, oral prednisone was initiated at 20 mg daily, with a weekly taper of 5 mg. However, 12 days after discharge, she was readmitted with abdominal distension. At that time, she also presented with hypotension and ophthalmoplegia. Brain MRI revealed abnormal signals in the medial and lateral rectus muscles bilaterally. Echocardiography revealed a reduced left ventricular ejection fraction (LVEF) of 38%. Endomyocardial biopsy confirmed lymphocytic myocarditis, and serology was positive for anti-SSA/Ro52 antibodies and borderline positive for anti-PL-7 antibody, leading to a diagnosis of UCTD-associated fulminant myocarditis. Treatment with methylprednisolone (60 mg daily) and IVIg resulted in clinical recovery. At three-year follow-up, the patient remained in remission, with complete resolution of diplopia, normalization of cardiac enzyme profile, and no progression to any definite connective tissue disease. This case suggests that isolated diplopia as the sole initial manifestation of UCTD-associated fulminant myocarditis is exceedingly rare. For autoimmune myocarditis, endomyocardial biopsy is crucial to guide treatment. Premature tapering of low-dose corticosteroids led to rapid relapse, highlighting the importance of starting with an adequate dose, tapering slowly, and maintaining the minimal effective dose.

## Introduction

Undifferentiated connective tissue disease (UCTD) is a term used to describe conditions characterized by signs and symptoms suggestive of a systemic autoimmune disorder that do not fulfill the classification criteria for any defined connective tissue disease (CTD), such as systemic lupus erythematosus (SLE), Sjögren's syndrome, rheumatoid arthritis, and others (1). UCTD is common, affecting 20%–52% of patients with connective tissue disease seen in rheumatology clinics, predominantly middle-aged women (1). Most patients remain stable over time, but approximately 28% evolve into a definite CTD (most often SLE or rheumatoid arthritis) within 5–6 years, while 18% achieve remission (2). Treatment typically involves low-dose glucocorticoids, hydroxychloroquine, or non-steroidal anti-inflammatory drugs; about one-third require immunosuppressants, and 10-year survival exceeds 90% (2). Nevertheless, formal clinical practice guidelines for UCTD are lacking, and both patients and clinicians face diagnostic and therapeutic uncertainty (1). UCTD can cause cardiovascular complications, among which fulminant myocarditis is the most critical. The clinical impact of autoimmune-related myocarditis is often underestimated because its symptoms overlap with those of more common cardiac diseases, and non-invasive, highly specific diagnostic tools are lacking (3). Furthermore, atypical presentations may lead to diagnostic delays with potentially fatal consequences. While acute myocarditis can occasionally present with ocular symptoms such as diplopia due to extraocular muscle involvement, isolated diplopia as the sole heralding manifestation of subsequent fulminant myocarditis in UCTD is exceedingly rare.

We report a case of biopsy-proven UCTD-associated fulminant myocarditis that first presented with isolated diplopia, initially leading to suspicion of a primary neurological disorder. The patient experienced relapse after premature tapering of low-dose corticosteroids, eventually progressing to cardiogenic shock. This case highlights the diagnostic challenges posed by atypical presentations, the critical role of endomyocardial biopsy in guiding treatment intensity, and the importance of adequate corticosteroid dosing and slow tapering in UCTD-associated myocarditis.

## Case report

### History of presentation in first visit

In May 2022, a 66-year-old Chinese woman, a farmer, presented to our hospital with a 17-day history of diplopia. The diplopia was exacerbated by upward gaze and was accompanied by blurred vision and intermittent throbbing pain. She denied any cardiopulmonary symptoms, including chest pain, palpitations, or syncope. Her medical history was unremarkable except for chronic gastritis; she had no history of hypertension, diabetes, or other systemic illnesses. Her personal and family histories were unremarkable. She had not received any immunomodulatory therapy prior to admission.

### Physical and neuro-ophthalmologic examination

On examination, her blood pressure was 105/70 mmHg, heart rate 71 beats/min, respiratory rate 11 breaths/min, and temperature 36.5 °C. 2103. Neurological examination showed bilateral ptosis and restricted adduction and abduction of the right eye (Figure 1A), with no other focal signs. Best-corrected visual acuity was 0.8 in both eyes. Pupils were equal, round, and reactive to light (3 mm bilaterally), with direct and consensual light reflexes present and no relative afferent pupillary defect. Ocular alignment was orthophoric in primary gaze. Extraocular motility: the right eye showed restricted adduction, abduction, and upgaze; the left eye showed restricted upgaze; downgaze and convergence were normal bilaterally. Diplopia testing revealed vertical diplopia on upgaze and right gaze, and horizontal diplopia on left and right gaze. Anterior segment and funduscopic examinations were unremarkable.

**Figure 1:**
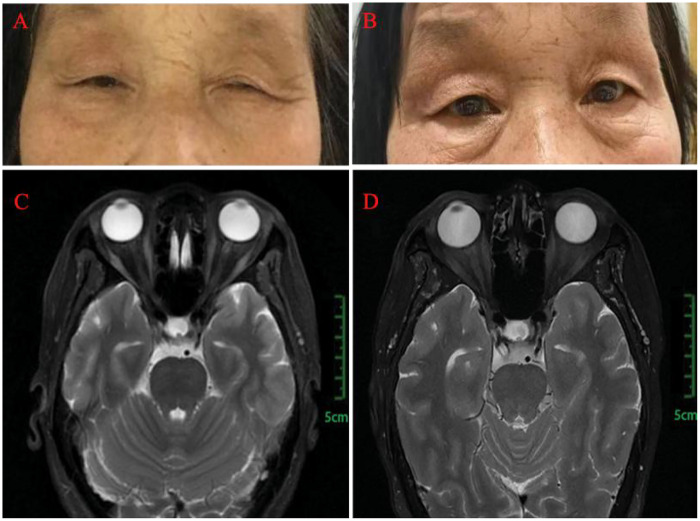
Clinical and MRI findings before and after treatment. **(A)** At the first visit, both eyes exhibited restricted upward gaze movements. **(B)** At three-year follow-up, the restricted eye movements had completely resolved. **(C)** At the first visit, localized T2WI hyperintensity was present in the bilateral medial and lateral rectus muscles. **(D)** At three-year follow-up, no abnormal signals were detected in the bilateral extraocular muscles.

### Laboratory tests and auxiliary examinations in first visit

Admission laboratory tests revealed the following abnormalities: plasma fibrinogen 4.70 g/L (reference range 2–4 g/L), erythrocyte sedimentation rate 59 mm/h (0–20 mm/h). Autoantibody profile: Homogeneous antinuclear antibody titer: 1:100 (<1:100); anti-dsDNA antibody (anti-dsDNA) 38.10 IU/mL (0–10 IU/mL); all other antibodies were within normal limits. Complement C3 was mildly decreased at 0.79 g/L (0.8–1.5 g/L). Cerebrospinal fluid analysis showed mildly elevated protein (0.43 g/L, reference range 0–0.4 g/L) and immunoglobulin G (56.40 mg/L, reference range 0–34 mg/L), with other cerebrospinal fluid parameters normal. Notably, myocardial injury markers were significantly elevated: creatine kinase (CK) 1507 U/L (40–200 U/L), creatine kinase-MB (CK-MB) 61.92 ng/mL (0–5 ng/mL), high-sensitivity troponin I (hs-cTnI) 1.4868 ng/mL (0–0.0175 ng/mL), B-type natriuretic peptide (BNP) 576 pg/mL (0–100 pg/mL), and myoglobin 258.60 ng/mL (1.5–70 ng/mL). A comprehensive panel of infectious, autoimmune, and neoplastic markers – including viral antibodies (Epstein–Barr virus, rubella, respiratory syncytial virus, adenovirus, cytomegalovirus), tumor markers, complement C4, vasculitis panel, quantitative immunoglobulin, and anti-ganglioside antibodies – was unremarkable. Serum cholinesterase was normal, and acetylcholine receptor antibodies were negative. Thrombophilia screening (lupus anticoagulant, protein S, protein C, antithrombin III) was unremarkable. Plain and contrast-enhanced magnetic resonance imaging (MRI) of the brain showed a few ischemic changes in the right frontal subcortical region. Electromyography revealed involvement of the sensory fibers of the bilateral median nerves.

### Hospitalization diagnosis and treatment process in first visit

The neurology department initially diagnosed Miller-Fisher syndrome and administered four sessions of plasma exchange. Admission electrocardiogram showed acute anterior wall myocardial infarction-like changes with premature ventricular contractions. Emergency coronary angiography was performed due to suspicion of acute myocardial infarction, which revealed no coronary artery stenosis. Subsequently, the patient developed sustained refractory ventricular tachycardia, which was terminated by intravenous lidocaine. On hospital day 8, bedside transthoracic echocardiography showed impaired left ventricular diastolic function and a left ventricular ejection fraction (LVEF) of approximately 53%. Cardiac MRI demonstrated diffuse elevated T1 mapping signal in the left ventricular myocardium, elevated T2 mapping signal in the basal anterior and anterior septal walls, and subendocardial delayed enhancement (Figures 2A–C). The atypical presentation of isolated diplopia without cardiac symptoms posed an initial diagnostic challenge, delaying recognition of myocarditis.

**Figure 2:**
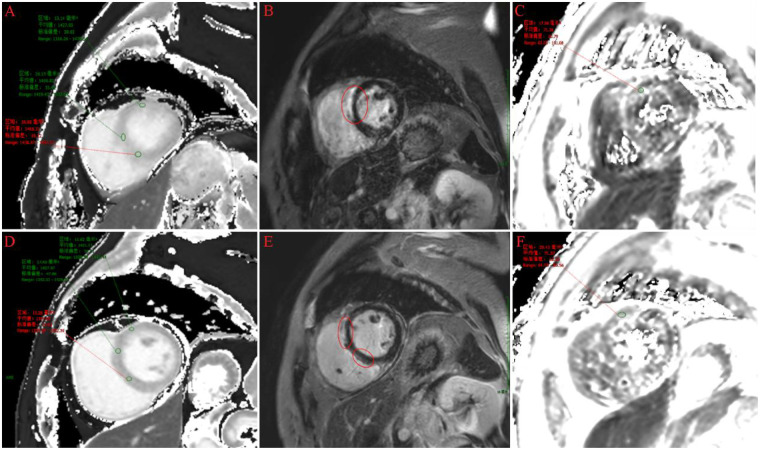
Cardiac MRI findings at first and second admissions. At first admission: **(A)** Elevated T1 mapping signal in the myocardium. **(B)** Mild delayed enhancement in the myocardium. **(C)** Elevated T2 mapping signal in the myocardium. At second admission: **(D)** Markedly elevated T1 mapping signal in the myocardium. **(E)** Significant delayed enhancement in the myocardium. **(F)** Markedly elevated T2 mapping signal in the myocardium.

Because of persistently rising myoglobin and troponin levels together with the cardiac MRI findings, the cardiology department diagnosed acute myocarditis. Treatment was initiated with methylprednisolone (20 mg once daily) combined with intravenous immunoglobulin (IVIg) (10 g once daily), with continuous monitoring of cardiac enzyme profile and biomarkers. The patient's ophthalmoplegia gradually improved, and the myocardial enzyme profile showed a significant decrease. CK-MB and hs-cTnI decreased to 25 ng/mL and 0.0557 ng/mL, respectively. She was discharged on day 23 of hospitalization. Upon discharge, oral prednisone (20 mg once daily) was prescribed, with a weekly taper of 5 mg.

### History of presentation in second visit

Ten days after discharge, the patient developed acute diarrhea of unclear etiology. After the diarrhea resolved, her diplopia worsened, accompanied by abdominal distension and constipation. Two days after symptom deterioration, she presented to our hospital on June 20, 2022. On admission, she was in cardiogenic shock. Physical examination showed blood pressure 81/47 mmHg, right-sided ptosis, restricted upward gaze, adduction, and abduction of the right eye, and restricted upward gaze of the left eye; no other abnormalities were noted.

### Laboratory tests and auxiliary examinations in second visit

Laboratory findings showed the following abnormalities: D-dimer 1.35 mg/L (0–0.5 mg/L), C-reactive protein 91.76 mg/L (0–6 mg/L), CK 5582 U/L (40–200 U/L), CK-MB 209.3 ng/mL (0–5 ng/mL), anti-dsDNA 15.25 IU/mL (0–10 IU/mL), and erythrocyte sedimentation rate 60 mm/h (0–20 mm/h). Semi-quantitative testing of the 24-item myositis autoantibody panel showed positivity only for anti-SSA/Ro52 (30 AU), borderline for anti-PL-7 (9 AU), and negativity for all remaining antibodies. Re-review of all initial admission findings revealed that localized T2WI hyperintensity in the bilateral medial and lateral rectus muscles had already been present at that time (Figure 1C); however, this finding was overlooked, resulting in misdiagnosis of the etiology of diplopia from unrecognized extraocular muscle involvement.

Follow-up cardiac MRI demonstrated findings consistent with inflammatory cardiomyopathy, with reduced left ventricular systolic function and an LVEF of 44.4%. Bedside echocardiography showed markedly reduced wall motion in the inferior wall, inferoseptal wall, and posterior wall, with reduced left ventricular systolic function and an LVEF of 38%. To clarify the myocardial pathology, percutaneous endomyocardial biopsy was performed, and myocardial tissue was obtained from the right ventricular septum. Light microscopy showed diffuse infiltration of the myocardium by CD3⁺, CD4⁺, CD8⁺, CD20⁺, and CD68⁺ lymphocytes, accompanied by myocardial fibrosis. No eosinophils or giant cells were identified. These findings were consistent with acute lymphocytic myocarditis. Transmission electron microscopy revealed regularly arranged myofibrils with focal dissolution. Mitochondria were sparse, without cristae disruption or lysis. No viral particles, glycogen accumulation, or myelin-like structures were observed within cardiomyocytes. The basal lamina of myocytes was intact. The interstitium showed infiltration by lymphocytes, macrophages, and plasma cells, along with marked proliferation of collagen fibers (Figure 3).

**Figure 3:**
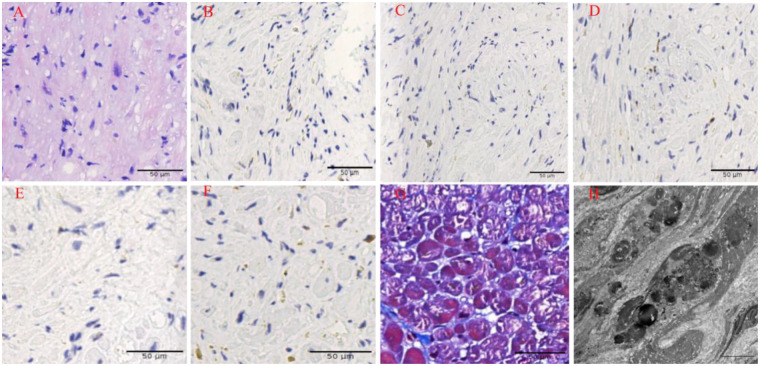
Pathological examination results of myocardial tissue in the patient. **(A)** HE staining (×20) reveals intracellular vacuoles in myocardial cells and infiltration of lymphocytes. **(B)** Immunohistochemical staining for CD3⁺ (×20). **(C)** Immunohistochemical staining for CD4⁺ (×20). **(D)** Immunohistochemical staining for CD8⁺ (×20). **(E)** Immunohistochemical staining for CD20⁺ (×20). **(F)** Immunohistochemical staining for CD68⁺ (×20). **(G)** Masson's trichrome staining showing myocardial fibrosis (×20). **(H)** Transmission electron microscopy (×5000).

### Management in second visit

The relapse was attributed to insufficient corticosteroid dosage during the first admission. Treatment was therefore intensified to methylprednisolone (60 mg once daily) combined with IVIg (10 g once daily). After adequate treatment, the patient's cardiac function returned to normal, with LVEF increasing to 52%. CK-MB, hs-cTnI, and myoglobin dropped to 8.2 ng/mL, 0.033 ng/mL, and 66.7 ng/mL, respectively. The patient was discharged on July 5, 2022. After discharge, oral prednisone was prescribed at 60 mg/d, tapered by 5 mg every two weeks to a maintenance dose of 5 mg/d. A slower tapering regimen was chosen based on the lesson learned from the rapid relapse after premature dose reduction.

### Subsequent treatment and follow-up

The patient was followed regularly. One month after second discharge, CK-MB was 7.8 ng/mL. At two months, CK-MB decreased to 6.90 ng/mL, and prednisone was continued to be tapered, eventually to a maintenance dose of 5 mg/d. At three years after discharge, the patient's diplopia had completely resolved (Figure 1B). Apart from mild weight gain of approximately 2 kg, the patient had no long-term steroid-related side effects such as central obesity or elevated blood glucose. Her condition remained stable without progression to any definite CTD. Laboratory tests showed anti-dsDNA 15.73 IU/mL (0–10 IU/mL), anti-histone antibody 23.32 RU/mL (0–20 RU/mL), and BNP 239 pg/mL (0–100 pg/mL); cardiac enzyme profile was normal. Echocardiography revealed an LVEF of 52%. Brain MRI showed no abnormal signal in the bilateral extraocular muscles (Figure 1D). Because anti-dsDNA and anti-histone antibodies remained mildly elevated, the patient continued oral maintenance therapy with low-dose prednisone acetate 5 mg/d. The comprehensive timeline of both admissions and the three-year follow-up is presented in Table 1.

**Table 1:** Timeline.

Time	Event/Treatment	Cardiac, laboratory, and immune parameters
2022-05-17 (T0) First admission	Initial presentation: diplopia, ophthalmoplegia (Figure 1A); Brain MRI: a few ischemic changes in right frontal subcortical region.	CK:1,507.00 U/L; CK-MB:61.92 ng/mL; hs-cTnI:1.49 ng/mL; myoglobin:258.60 ng/mL; BNP:576.00 pg/mL; LVEF:53.00%
2022-05-18 (T0 + 1d)	Coronary angiography: no stenosis; First plasma exchange.	CK:671.00 U/L; CK-MB:51.06 ng/mL; hs-cTnI:0.34 ng/mL; myoglobin:73.70 ng/mL; BNP:1,241.72 pg/mL; K⁺:5.44 mmol/L; Anti-dsDNA:38.10 IU/mL
2022-05-24 (T0 + 7d)	Fourth plasma exchange; Complication: sustained ventricular tachycardia (terminated by lidocaine).	CK:1,167.00 U/L; CK-MB:55.88 ng/mL; hs-cTnI:2.95 ng/mL; myoglobin:73.70 ng/mL; BNP:1,241.72 pg/mL
2022-05-27 (T0 + 10d)	Cardiac MRI: consistent with inflammatory cardiomyopathy (Figures 2A–C); First immunotherapy: methylprednisolone 20 mg/d + IVIg 10 g/d initiated.	CK:339.00 U/L; CK-MB:37.92 ng/mL; hs-cTnI:2.17 ng/mL; myoglobin:35.99 ng/mL; BNP:1,586.00 pg/mL
2022-06-08 (T0 + 22d) First discharge	Discharged on prednisone 20 mg/d, weekly taper by 5 mg.	CK:127.00 U/L; hs-cTnI:0.06 ng/mL; myoglobin:66.70 ng/mL; BNP:475.00 pg/mL
2022-06-20 (T0 + 34d) Second admission	Cardiogenic shock; Diplopia worsened; Treatment intensified: methylprednisolone 60 mg/d + IVIg 10 g/d.	CK:5,582.00 U/L; CK-MB:209.30 ng/mL; hs-cTnI:13.16 ng/mL; myoglobin:572.10 ng/mL; BNP:2,950.00 pg/mL; LVEF:38%; Anti-dsDNA:15.25 IU/mL
2022-06-23 (T0 + 37d)	Re-review of initial brain MRI: localized T2WI hyperintensity in bilateral medial and lateral rectus muscles (Figure 1C); Repeat cardiac MRI: consistent with inflammatory cardiomyopathy, reduced LV systolic function, LVEF 44.4% (Figures 2D–F).	CK-MB:42.69 ng/mL
2022-06-27 (T0 + 41d)	Endomyocardial biopsy: acute lymphocytic myocarditis; Electron microscopy: no viral particles (Figure 3).	CK:1,476.00 U/L; CK-MB:19.00 ng/mL; hs-cTnI:7.50 ng/mL; myoglobin:127.00 ng/mL; BNP:2,110.00 pg/mL; Anti-SSA/Ro52:30 AU; Anti-PL-7:9 AU (borderline)
2022-07-04 (T0 + 48d) Second discharge	Discharged on prednisone 60 mg/d, slow taper (5 mg every 2 weeks) to maintenance dose of 5 mg/d.	CK:38.00 U/L; CK-MB:8.20 ng/mL; hs-cTnI:0.03 ng/mL; myoglobin:66.70 ng/mL; BNP:239.00 pg/mL; LVEF:52.00%
2025 (Three-year follow-up)	Diplopia completely resolved (Figure 1B); No progression to definite CTD; Brain MRI: no abnormal signal in bilateral extraocular muscles (Figure 1D).	normal cardiac enzyme profile; BNP 239.00 pg/mL; LVEF 52.00%; Anti-dsDNA:15.73 IU/mL; Anti-histone:23.32 RU/mL

CK, creatine kinase; CK-MB, creatine kinase-MB; hs-cTnI, high-sensitivity cardiac troponin I; BNP, B-type natriuretic peptide; LVEF, left ventricular ejection fraction; IVIg, intravenous immunoglobulin; CTD, connective tissue disease. All numerical values in the table are presented with two decimal places. Missing values indicate test not performed or result not reported. Anti-U1-RNP antibody was not tested due to laboratory constraints.

## Discussion

This report describes a rare case of UCTD-related fulminant myocarditis that initially presented with isolated diplopia, posing a significant diagnostic challenge. The differential diagnosis included the following conditions. We first considered Miller-Fisher syndrome. It is a rare subtype of Guillain-Barré syndrome, which is an inflammatory demyelinating neuropathy. This disorder typically presents with the triad of ophthalmoplegia, ataxia, and areflexia, often accompanied by anti-GQ1b antibodies (4). Our patient had diplopia and mildly elevated cerebrospinal fluid protein, but she never developed ataxia or areflexia, and anti-GQ1b antibodies were negative. Thus, Miller-Fisher syndrome was excluded. Next, we needed to rule out ocular myasthenia gravis. This disease is mediated by acetylcholine receptor antibodies and is characterized by fluctuating, fatigable ophthalmoplegia with diurnal variation; 30%–77% of patients test positive for acetylcholine receptor antibodies (5). Our patient's diplopia lacked clear diurnal fluctuation, acetylcholine receptor antibodies were negative, and the severe myocardial injury could not be explained by this disorder. Therefore, ocular myasthenia gravis was not supported. Antisynthetase syndrome required further differentiation. This syndrome is diagnosed by the presence of antisynthetase antibodies together with at least one of myositis, interstitial lung disease, or arthritis; mechanic's hands and Raynaud phenomenon are supportive (6). Our patient had only a borderline anti-PL-7 antibody, no myalgia or proximal muscle weakness, no interstitial lung disease, no arthritis, and no typical cutaneous lesions. Hence, the diagnosis was not met. The significantly elevated CK and myoglobin levels in this patient prompted consideration of polymyositis/dermatomyositis. Polymyositis is an autoimmune chronic inflammatory myopathy characterized by symmetric proximal muscle weakness due to endomysial inflammation, while dermatomyositis involves the perimysium and presents with typical skin manifestations (Gottron's papules/sign, heliotrope rash) (7). Anti-Jo-1 antibody, which is positive in approximately 25% of polymyositis cases, is a specific marker. Nearly all patients with polymyositis have abnormal electromyographic findings, including changes in amplitude and velocity of membrane action potentials, as well as fibrillation potentials due to membrane irritability (7). In the present case, the absence of proximal limb weakness, heliotrope rash or Gottron's papules, along with no myopathic changes on electromyography, argues against polymyositis/dermatomyositis. According to the 2019 European League Against Rheumatism/American College of Rheumatology classification criteria, a positive antinuclear antibody (≥1:80) is required as an entry criterion, and weighted points are assigned across clinical and immunological domains, with a total score of ≥10 needed for classification (8). Our patient had antinuclear antibody at 1:100 and anti-double-stranded DNA at 38.10 IU/mL, but lacked sufficient clinical points to reach the threshold of 10. Hence, systemic lupus erythematosus was not established. Mixed connective tissue disease is a rare systemic autoimmune disorder characterized by overlapping manifestations of at least two CTDs among SLE, systemic sclerosis, polymyositis, and rheumatoid arthritis. It is defined by a high titre of anti-U1-ribonucleoprotein antibody as the core serological feature, with typical clinical manifestations including Raynaud phenomenon, swollen hands, arthritis, and myositis (9). Although anti-U1-RNP antibodies were not tested in this patient, her clinical manifestations did not fulfill the characteristic features of the disease. In summary, this patient presented with extraocular muscle and myocardial involvement, along with laboratory abnormalities including antinuclear antibodies and anti-dsDNA autoantibodies, suggesting an underlying CTD. However, she did not meet the diagnostic criteria for well-defined CTDs, and was therefore classified as having UCTD. UCTD was considered to be responsible for the patient's extraocular myositis and myocarditis, ultimately leading to diplopia and the clinical presentation of fulminant myocarditis.

Although no universally accepted classification criteria exist for UCTD, the most widely recognized definition, proposed by Mosca et al., requires the presence of at least one clinical manifestation suggestive of a CTD, a positive antinuclear antibody on at least two occasions, and the absence of criteria for any definite CTD (1, 2). A disease duration cutoff of three years has been used to differentiate between “evolving UCTD” and “stable UCTD” (1). According to a retrospective study of 145 patients with UCTD, diagnosis can be considered after more than two years of symptoms or the presence of high-titer non-organ-specific autoantibodies in the absence of definite CTD criteria (10). During the three-year follow-up period, the patient remained stable with no evidence of disease evolution, which is consistent with the clinical features of stable UCTD.

Furthermore, the most striking feature of this case is the simultaneous involvement of both the extraocular and cardiac muscles, but the underlying mechanism remains speculative. Autoantibodies, particularly anti-SSA/Ro52, have been reported to be associated with autoimmune myocarditis (11, 12). Because both the myocardium and extraocular muscles belong to striated muscle and may share common antigenic components, we hypothesize that anti-SSA/Ro52 might cross-react with these two tissues, triggering local inflammation. However, direct evidence is lacking, as an extraocular muscle biopsy was not performed. Therefore, the precise pathway from isolated diplopia to fulminant myocarditis in this UCTD patient remains speculative and requires further investigation.

The etiology of fulminant myocarditis includes infectious and non-infectious factors. Viral infection is the main cause. However, only 10%–20% of patients with viral myocarditis have positive virological testing in myocardial tissue (13). Endomyocardial biopsy is not only the gold standard for diagnosing myocarditis, but can also identify and quantify pathogens such as viruses (14). More importantly, it can guide treatment intensity. Based on the type of infiltrating inflammatory cells, fulminant myocarditis is classified pathologically as lymphocytic, eosinophilic, or giant cell myocarditis, with giant cell myocarditis having the worst prognosis (13). Immune overactivation and inflammatory storms cause severe myocardial injury. Treatment with adequate doses of corticosteroids combined with immunoglobulin can block the pathogenic mechanisms, reduce inflammation and edema, counteract shock, alleviate clinical symptoms, save lives, and improve prognosis. In this patient, transmission electron microscopy of myocardial tissue revealed no viral particles, and the underlying etiology was UCTD. Endomyocardial biopsy confirmed lymphocytic myocarditis. Therefore, the optimal treatment was intensive immunosuppressive therapy with adequate doses of methylprednisolone combined with intravenous immunoglobulin rather than high-dose steroid pulse therapy alone.

Endomyocardial biopsy remains the gold standard for diagnosing myocarditis and, more importantly, for guiding treatment intensity (11). After the first discharge, severe insufficiency of corticosteroid dosage led to clinical deterioration and fulminant myocarditis within just 10 days. In contrast, after the second discharge, the patient received an adequate oral dose of prednisone acetate (60 mg/d), tapered by 5 mg every two weeks, and maintained on a long-term dose of 5 mg/d to prevent relapse. To date, the patient has remained stable. These observations underscore that in autoimmune myocarditis due to active UCTD, an adequate initial dose and a gradual tapering regimen are crucial to avoid life-threatening deterioration. Although hydroxychloroquine is commonly used as baseline therapy for UCTD and has been shown to reduce cardiovascular events in autoimmune settings (12), it also carries well-documented cardiotoxicity, including QT prolongation, myocardial fibrosis, and conduction blockades, particularly at higher doses or with long-term use (15). Given that this patient had already experienced fulminant myocarditis with significant myocardial injury, the additional cardiac toxicity risk was considered unacceptable. Subsequently, we also considered discontinuing corticosteroids, but because the patient still had persistent mild autoimmune activity, we believe that the current maintenance dose of prednisone 5 mg/day represents the optimal balance between efficacy and safety.

Several limitations of this case report should be acknowledged. First, viral infection is a common cause of fulminant myocarditis. In this patient, given the absence of prodromal symptoms of viral infection, negative serum viral testing, and no viral particles detected on transmission electron microscopy of myocardial tissue, viral infection was considered unlikely; therefore, viral genomic analysis of the biopsy specimen was not performed. Second, due to laboratory constraints at our hospital, anti-U1-RNP antibody testing was not performed.

## Conclusion

This case suggests that adequate dosage and full course of corticosteroid therapy is essential for UCTD-associated fulminant myocarditis presenting with isolated diplopia. In UCTD patients with persistently mild antinuclear antibody and anti-dsDNA elevation, long-term low-dose prednisone acetate (5 mg/d) maintenance therapy plays a key role in preventing recurrence of myocarditis and extraocular myositis. No significant steroid-related side effects were observed during long-term follow-up.

## Patient perspective

“At the beginning, my condition kept relapsing and getting worse, and the doctors couldn't figure out what was wrong. I felt so pessimistic and desperate – it was devastating both physically and mentally. But the medical team worked tirelessly, finally made a clear diagnosis, tailored a treatment plan for me, and even gave me rehabilitation guidance after I left the hospital. Now I am completely cured and haven't had a relapse. I am truly grateful to this medical team. They cured my disease and helped me get back to normal life and enjoy every day.”

## Data Availability

The original contributions presented in the study are included in the article/supplementary material, further inquiries can be directed to the corresponding authors.
